# Small and Large Seminal Extracellular Vesicles Differentially Modulate Capacitation‐Related Parameters in Frozen–Thawed Porcine Epididymal Spermatozoa

**DOI:** 10.1111/rda.70222

**Published:** 2026-09-30

**Authors:** Patricia Panales, Junwei Li, Ana Parra, Pablo Martínez‐Díaz, Xiomara Lucas, Marc Yeste, Jordi Roca, Isabel Barranco

**Affiliations:** ^1^ Department of Medicine and Animal Surgery, Faculty of Veterinary Science University of Murcia Murcia Spain; ^2^ International Excellence Campus for Higher Education and Research Campus Mare Nostrum, Institute for Biomedical Research of Murcia (IMIB‐Arrixaca), University of Murcia Murcia Spain; ^3^ College of Veterinary Medicine, Yangzhou University Yangzhou China; ^4^ Biotechnology of Animal and Human Reproduction (TechnoSperm), Institute of Food and Agricultural Technology, University of Girona Girona Spain; ^5^ Unit of Cell Biology, Department of Biology, Faculty of Sciences University of Girona Girona Spain

**Keywords:** capacitation, epididymal spermatozoa, extracellular vesicles, pig, seminal plasma

## Abstract

Seminal extracellular vesicles (sEVs) have been proposed as modulators of sperm function, but their exact role remains unclear. Most studies use ejaculated sperm for clarification. This approach has limitations; nevertheless, as ejaculated sperm have already been exposed to their own native sEVs, which hinders the assessment of their specific roles. This study aimed to evaluate the potential impact of small (S‐) and large (L‐) sEVs on capacitation‐related parameters of epididymal porcine spermatozoa. Frozen–thawed epididymal sperm were incubated in a non‐capacitating medium with or without S‐ or L‐sEVs at 37°C under 5% CO_2_ for 3 h. Acrosome integrity, membrane lipid disorder, intracellular Ca^2+^ and protein tyrosine phosphorylation (pTyr) were assessed by flow cytometry at 30 min and 3 h of incubation. Both S‐sEVs and L‐sEVs decreased (*p* < 0.0001) membrane lipid disorder at 30 min, but not at 3 h. L‐sEVs increased (*p* < 0.05) intracellular Ca^2+^ levels at 30 min and decreased (*p* < 0.01) pTyr at 3 h. These results demonstrate a differential, time‐dependent modulation of capacitation‐related parameters by S‐ and L‐sEVs in porcine epididymal spermatozoa. This provides direct evidence of their primary effects on spermatozoa not exposed to seminal plasma.

## 1 Introduction

Seminal plasma (SP), a protein‐rich fluid produced by the testes, the epididymis and the accessory sex glands, plays a key role in modulating sperm function (Rodriguez‐Martinez et al. 2021). The SP contains extracellular vesicles (sEVs), which are released by most functional cells of the male reproductive tract (Roca et al. 2022). These nanosized particles are known to carry bioactive molecules, such as proteins, lipids, metabolites and nucleic acids, to spermatozoa and functional cells of the female reproductive tract, modulating their functionality (Roca et al. 2022). Studies evaluating the impact of sEVs on sperm function have produced conflicting results, particularly concerning their role in sperm capacitation. These studies used ejaculated sperm that had already been exposed to sEVs during and after ejaculation (Martínez‐Díaz et al. 2024). This makes it difficult to determine the initial impact of sEVs. Therefore, epididymal sperm may be a more suitable model for investigating the specific functional role of sEVs on sperm because they have not previously been exposed to SP. Accordingly, this study evaluated the potential effects of two sEV subsets, small (S‐sEVs) and large (L‐sEVs), on capacitation‐related parameters of porcine epididymal spermatozoa.

## 2 Materials and Methods

### 2.1 Boars, Epididymal Sperm and Seminal Plasma Samples

Ejaculates and epididymal sperm samples were collected from healthy, fertile boars used in artificial insemination (AI) programs (AIM Iberica, Topics Norsvin, Madrid, Spain). Epididymal sperm were collected *postmortem* and cryopreserved using a standard 0.5‐mL straw protocol (Alkmin et al. 2014). SP samples were collected after two sequential centrifugations of the ejaculate samples (1500 × *g* for 10 min) and stored at −80°C until sEV isolation.

### 2.2 Isolation and Characterization of sEVs

Seminal EV subsets (S‐sEVs and L‐sEVs) were isolated from SP samples using a size‐exclusion chromatography (SEC)‐based procedure described by (Martínez‐Díaz et al. 2025). The sEV samples were characterized using complementary approaches, following the recommendations recorded in the minimal information for studies of extracellular vesicles (MISEV) guidelines (Welsh et al. 2024) and according to MIFlowCyt guidelines (Lee et al. 2008).

### 2.3 Sperm Functionality Assessments

Sperm capacitation‐related parameters were analysed using a CytoFlex flow cytometer (Beckman Coulter; Brea, CA, USA) following the protocols of Garriga et al. 2026 and (Peris‐Frau et al. 2019). Fluorochromes were purchased from Thermo Fisher Scientific (Waltham, MA, USA) and Abcam (Cambridge, UK). The analysed sperm parameters were acrosome integrity (PNA‐FITC/PI), membrane lipid disorder (M540/Yo‐Pro‐1), intracellular Ca^2+^ levels (Fluo4‐AM/PI) and tyrosine phosphorylation (pTyr; antibody conjugated with AlexaFluor647). Results were recorded as the percentage of viable sperm with an intact acrosome (PNA‐FITC^−^/PI^−^), the mean fluorescence intensity (MFI) of Fluo4‐AM and M540 in viable sperm (PI^−^ or Yo‐Pro‐1^−^, respectively), and the MFI of pTyr in the total sperm population.

### 2.4 Experimental Design

Frozen–thawed epididymal sperm samples were diluted to 50 × 10^6^ spermatozoa/mL in Beltsville Thawing Solution (BTS). The samples were supplemented or not (control) with S‐sEVs or L‐sEVs (0.2 mg/mL total protein) and incubated at 37°C under 5% CO_2_ for 3 h. Sperm capacitation‐related parameters were assessed at 30 min and 3 h of incubation. Five biological replicates were examined. The results were normalized to the control for each replicate and time point.

### 2.5 Statistical Analysis

Analyses were performed using GraphPad Prism 10.5.0 and IBM SPSS 29.0. Normal distribution and homoscedasticity were evaluated with the Shapiro–Wilk and Levene tests. Differences in sperm parameters among experimental groups were analysed using a linear mixed model and Sidak's post hoc test. Statistical significance was set at *p* ≤ 0.05.

## 3 Results

Although neither sEV subset affected the percentage of viable sperm with intact acrosome (Figure 1A), both sEV subsets lowered (*p* < 0.0001) membrane lipid disorder after 30 min of incubation but not after 3 h (Figure 1B). Intracellular Ca^2+^ levels were higher (*p* < 0.05) only in samples incubated with L‐sEVs for 30 min, but not after 3 h (Figure 2A). Finally, pTyr levels were lower (*p* < 0.01) only in sperm incubated with L‐sEVs for 3 h (Figure 2B).

**FIGURE 1:**
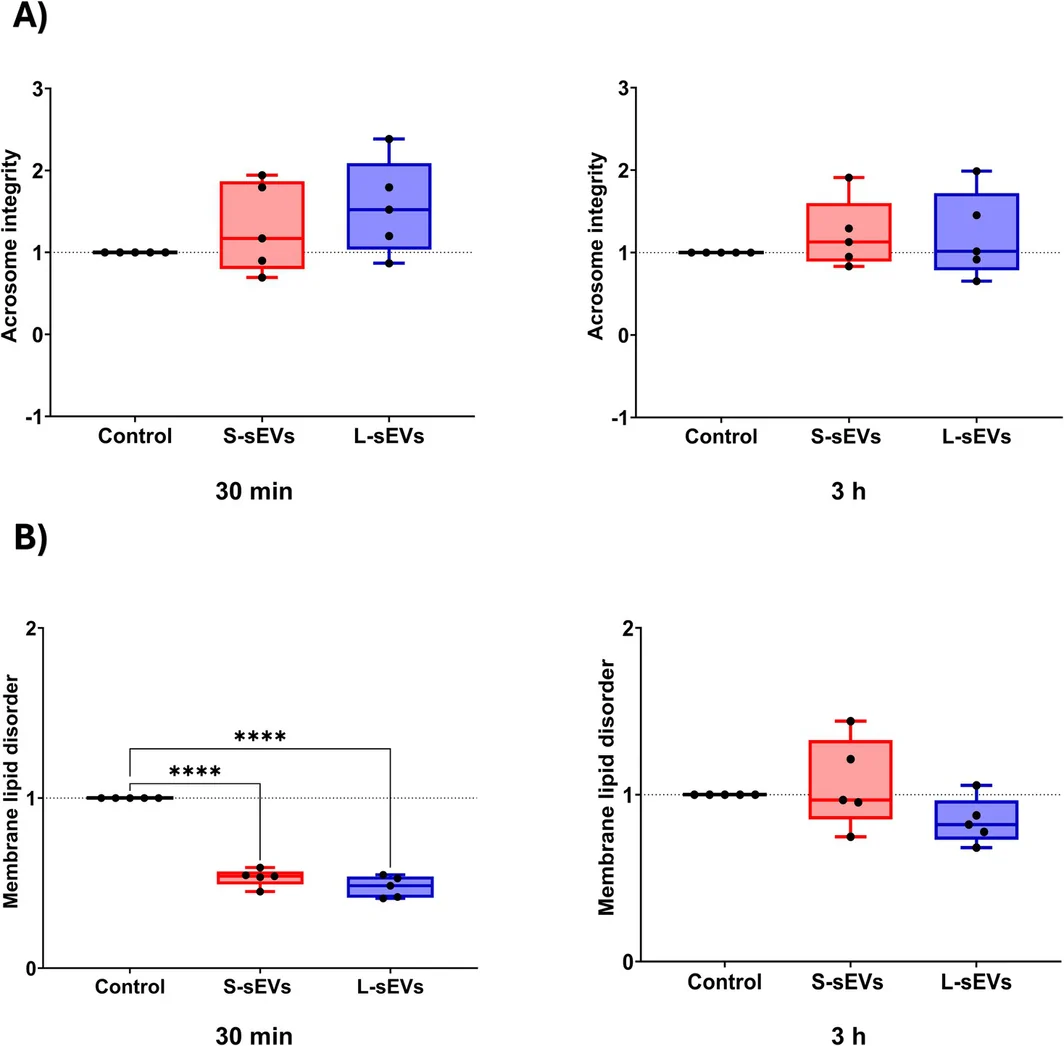
Effect of small (S‐) and large (L‐) seminal extracellular vesicles (sEVs) on (A) acrosome integrity and (B) membrane lipid disorder of frozen–thawed porcine epididymal spermatozoa after 30 min and 3 h of incubation at 37°C under 5% CO_2_. The results were normalized to the control group (without sEVs). (****) indicates differences between treatments at *p* < 0.0001.

**FIGURE 2:**
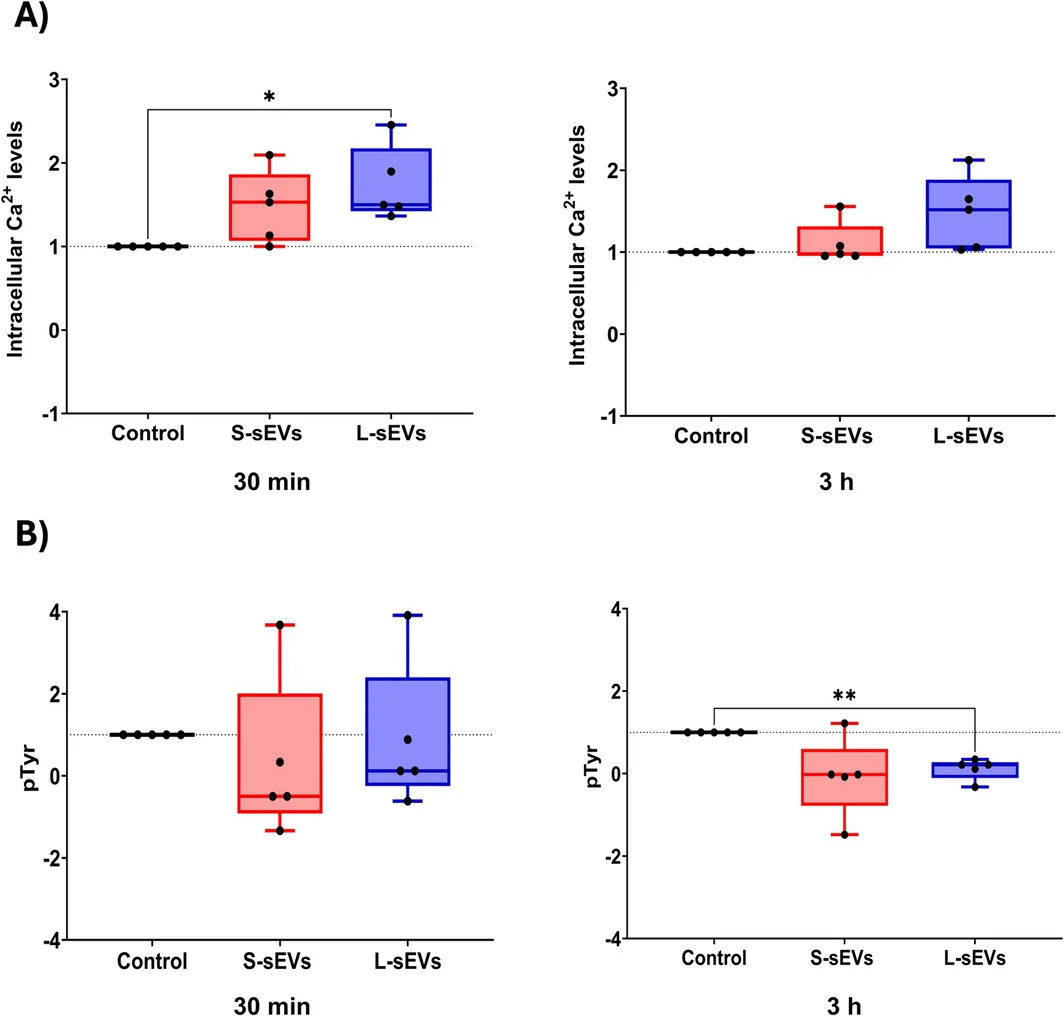
Effect of small (S‐) and large (L‐) seminal extracellular vesicles (sEVs) on (A) intracellular Ca^2+^ and (B) tyrosine phosphorylation (pTyr) levels in frozen–thawed porcine epididymal spermatozoa after 30 min and 3 h of incubation at 37°C under 5% CO_2_. The results were normalized to the control group (without sEVs). (*) and (**) indicate differences at *p* < 0.05 and *p* < 0.01, respectively.

## 4 Discussion

Neither S‐sEVs nor L‐sEVs affected acrosome integrity, suggesting that sEVs do not cause premature acrosome exocytosis. This finding is consistent with human studies (Tamessar et al. 2024) and is physiologically relevant because premature acrosome reaction is a hallmark of sperm dysfunction and reduced fertility. The transient decrease in membrane lipid disorder caused by both sEV subsets suggested an early sperm membrane‐stabilizing effect. This finding aligns with the results of Du et al. 2016 and supports the role of SP in preventing premature sperm capacitation (Roca et al. 2022).

Another interesting finding is the selective activation of intracellular signalling by L‐sEVs. This is characterized by a transient increase in intracellular Ca^2+^, followed by a reduction in pTyr. Although both processes are typically linked to capacitation‐related signalling, the modulation by L‐sEVs differs from the standard capacitation pattern. The early rise in Ca^2+^ is likely a direct consequence of sEV‐mediated Ca^2+^ transfer rather than the activation of endogenous capacitation signalling pathways. Conversely, the decrease in pTyr after 3 h of incubation suggests that L‐sEVs attenuate sperm capacitation. The results agree with those of Moya‐Fernández et al. (2025), which support the theory that L‐sEVs are fine regulators of sperm signalling rather than direct inducers of sperm capacitation.

The differential effects of S‐ versus L‐sEVs could be related to differences in composition (Martínez‐Díaz et al. 2024) and support the idea that these sEV subtypes act as complementary modulators. The absence of Ca^2+^ or pTyr responses in the S‐sEV condition indicates sEVs primarily regulate early membrane activity, whereas L‐sEVs modulate downstream signalling. This duality may reconcile discrepancies in the literature, where whole sEVs have been reported to both promote (Murdica et al. 2019) and inhibit (Moya‐Fernández et al. 2025) sperm capacitation.

## 5 Conclusion

Although we cannot rule out the possibility that epididymal EVs or those derived from the egg yolk of the frozen extender influence sperm functionality, significant functional changes were observed in sperm from the treatment group (samples exposed to sEVs) compared to the control group (unexposed samples). These changes allow us to conclude that sEVs influence sperm capacitation. This effect varies depending on the sEV subset (S or L) and exposure duration. Both S‐ and L‐sEVs appear to modulate membrane lipid organization by reducing lipid disorder over time; however, only L‐sEVs activate intracellular Ca^2+^ signalling and inhibit pTyr. These results suggest that sEVs play a role in regulating capacitation‐associated pathways without causing premature membrane destabilization or acrosome exocytosis.

## Author Contributions

P.P. and J.L.: writing, visualization, methodology, investigation. A.P. and P.M.‐D.: visualization, methodology, investigation. X.L., M.Y., J.R. and I.B.: writing – review and editing, validation, supervision, resources, funding acquisition, formal analysis, conceptualization.

## Funding

The study was supported by: (1) Ministerio de Ciencia, Innovación y Universidades (MICIU/AEI/10.13039/501100011033) and ERDF/EU (PID2022‐137738NA‐I00, PID2023‐149853OB‐I00 and PID2024‐159343OB‐I00), (2) MICIU/AEI/10.13039/501100011033 and European Union NextGenerationEU/PRTR program (RYC2021‐034546‐I), and Fundación Séneca (FSRM/10.13039/100007801) (21935/PI/22, 22784/FPI/24 and 23014/GERM/25).

## Ethics Statement

The Bioethics Committee of the University of Murcia approved the experiment (research code CBE 538/2023 and CBE 668/2025).

## Conflicts of Interest

The authors declare no conflicts of interest.

## Data Availability

Data supporting the findings of this study are available from the corresponding author upon request.
